# Development of a Chemically Defined Medium for *in vitro* Expansion of Primary Bovine Satellite Cells

**DOI:** 10.3389/fbioe.2022.895289

**Published:** 2022-08-04

**Authors:** Anna M. Kolkmann, Anon Van Essen, Mark J. Post, Panagiota Moutsatsou

**Affiliations:** ^1^ Mosa Meat BV, Maastricht, Netherlands; ^2^ Department of Physiology, Maastricht University, Maastricht, Netherlands

**Keywords:** cultured meat, serum-free medium, animal free medium, medium development, myoblasts, satellite cells

## Abstract

The use of fetal bovine serum (FBS) in animal cell culture media is widely spread since it provides a broad spectrum of molecules that are known to support cell attachment and growth. However, the harvest and collection procedures of FBS raise ethical concerns and serum is an ill-defined and expensive component. This is especially problematic when it comes to regulatory approval for food applications like cultured meat. The aim of this study is to develop a chemically defined, cost efficient serum-free and animal-free medium that supports the attachment and expansion of bovine myoblasts while maintaining their differentiation capacity. Bovine satellite cells were harvested and isolated from a fresh sample of skeletal muscle tissue and cultured in planar systems. The efficacy of the tested formulations was assessed with metabolic assays and cell counting techniques. Optical microscopy was used to observe cellular morphology and statistical analysis was applied. Based on a comprehensive literature analysis, a defined serum-free medium (SFM) composition was developed consisting of DMEM/F12 as basal medium, supplemented with L-ascorbic acid 2-phosphate, fibronectin, hydrocortisone, GlutaMAX^™^, albumin, ITS-X, hIL-6, α-linolenic acid, and growth factors such as FGF-2, VEGF, IGF-1, HGF, and PDGF-BB. To our knowledge, this is the first defined serum-free and animal free medium formulation specific for bovine myoblasts to date. We conclude that the SFM formulation supported exponential cell growth up to 97% of the serum—containing golden standard growth medium. All reagents used in this study are chemically defined.

## Introduction

With an expanding world population, rising affluence and urbanization, it is predicted that the consumption of meat will have increased by 70% in 2050 (FAO, 2006). Industrial animal farming and the consumption of animal products are the main reasons for global problems related to animal welfare, sustainability, and health. Meat production through livestock is a highly inefficient process, and the capacity of conventional meat production has nearly reached its maximum (McLeod, 2011). For these reasons, there is an urgent need for alternative meat production procedures. In this regard, one of the most promising technologies is cultured meat.

In 2013, our group provided the proof of principle that beef can be cultured using standard tissue engineering technology. However, the first *in vitro* hamburger only consisted of muscle tissue, and the culture medium contained animal-derived components which mostly came from the addition of fetal bovine serum (FBS).

On one hand, FBS is the most widely growth-promoting supplement in cell culture activities (Gstraunthaler, 2003). On the other hand, supplementing basal culture media with FBS raises ethical concerns due to the fact that it is obtained from the blood of bovine foetuses after pregnant cows have been slaughtered. In addition, it is poorly defined, is subject to lot-to-lot variability and is the most significant cost driver of cultured meat production. Lastly, if cultured meat results in a drastic reduction of livestock, the supply of fetal bovine serum will dry up.

For these reasons, efforts have been made to replace FBS [typically used in cell culture at 10% or 20% (v/v)] with a validated and cost-efficient animal-free medium supporting the attachment and growth of bovine satellite cells.

A specific transcriptional program is needed to activate cell growth and proliferation. Numerous growth factors, for example, Insulin-like growth factor 1 (IGF-1) and basic fibroblast growth factor (FGF-2), are present in FBS and activate cells in culture. Based on the pioneering serum free cell culture work of Sato et al., a basal medium has been developed that consists of 50% (v/v) Dulbecco’s Modified Eagle’s Medium (DMEM) and 50% (v/v) highly enriched HAM’s F-12 Nutrient Mixture (Barnes and Sato, 1980a; Barnes and Sato, 1980b). The basal medium needs to be supplemented with hormones, growth factors, vitamins, and other components that stimulate the proliferation of bovine satellite cells. Several growth factor receptors are known to be involved in regulating skeletal myogenesis and these include receptor tyrosine kinases, TGFR1, 2 & 3, patched, and wnt receptors (Sassoon, 2002).

Growth factors stimulate DNA synthesis in quiescent cells and prevent them from entering the G (0) state. Most growth factors stimulate mitogenic responses in a dose-dependent manner and often act synergistically with other growth factors or cytokines.

Unfortunately, the current offering in serum-free media for proliferation of bovine satellite cells is limited, and their performance is unsatisfactory (Miki and Takagi, 2015; Kolkmann et al., 2020). Therefore, the overall goal of this study is to develop a chemically defined serum-free medium that supports the attachment and expansion of bovine myoblasts while maintaining their differentiation capacity. A step-by-step methodology for developing such a medium is described herein. Overall, it is important that the final formulation meets the boundary requirements, namely simplicity, cost-efficiency and food-safety.

## Materials and Methods

### Materials

#### Serum-Containing Medium

Growth medium (GM) consisting of either Ham’s F-10 Nutrient Mix or DMEM/F-12 as basal medium, supplemented with 20% fetal FBS and 5 ng/ml FGF-2 was used as positive control in this study.

#### Experimental Design

**FIGURE 1:**
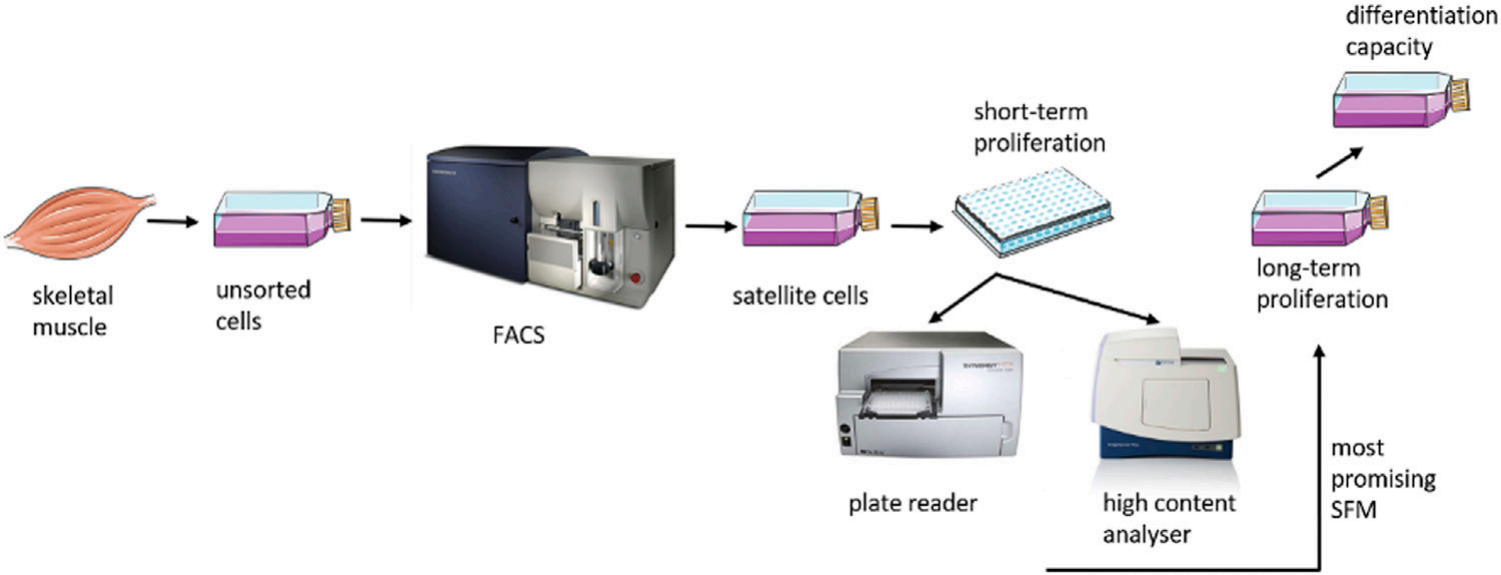
Schematic overview of the study.

The 5-step methodology used to develop the animal-free medium is described in Table 1 and the experimental setup is presented in Figure 1. Briefly, as a first step, the substitution of serum with single proteins was attempted based on stem cell literature. A relatively rich mix of components was used, including at least one basal medium, five of the most abundant components in serum, at least one mitogenic growth factor and at least two attachment factors. In step 2, the substitution of certain components with their animal-free homologues as well as the possibility to exclude some of them was evaluated in order to tailor it towards a cultured meat production application. In step 3, a two-level full factorial design of experiments was applied, to identify the optimal combination of growth factors associated with satellite cell proliferation. The results were analysed using the software JMP while GraphPad PRISM was used for graphical visualization. In step 4, the concentration of the costliest components was optimised and the resulting formulation was finally tested in step 5 as for its long-term performance and ability to maintain the cells’ differentiation capacity.

**TABLE 1:** Overview of the 5-step methodology for the animal-free medium development.

Step 1	Step 2 Substitution/elimination	Step 3 GF addition	Step 4 Concentration optimisation	Step 5Long term validation
Basal (F10)	Basal (F10 → DMEM/F12)	Basal (DMEM/F12)	Basal (DMEM/F12)	Basal (DMEM/F12)
Insulin	Insulin, transferrin, selenium → ITS-X	ITS-X	ITS-X	ITS-X
Transferrin				
Sodium selenite	BSA → HSA Fetuin Vitronectin	HSA Fetuin	HSA Fetuin	HSA
BSA Fetuin	Fibronectin	Fibronectin	Fibronectin	Fibronectin
Vitronectin	Somatotropin	Somatotropin	Somatotropin	
Fibronectin	Dexamethasone → Hydrocortisone	Hydrocortisone	Hydrocortisone	Hydrocortisone
Somatotropin	bIL-6 → hIL-6	hIL-6	hIL-6	hIL-6
Dexamethasone	α-linolenic acid	α-linolenic acid	α-linolenic acid	α-linolenic acid
bIL-6	FGF-2	FGF-2	FGF-2	FGF-2
α-linolenic acid	Asc-2-P	Asc-2-P	Asc-2-P	Asc-2-P
FGF-2	Heparan sulphate	Heparan sulphate	Heparan sulphate	
		IGF1	IGF1	IGF1
		LIF	LIF	
		PDGF-BB	PDGF-BB	PDGF-BB
		VEGF	VEGF	VEGF
		HGF	HGF	HGF

### Harvesting and Isolation of Cells

Bovine satellite cells were harvested and isolated from fresh samples of skeletal muscle tissue (*Bos taurus*) and sorted based on their positive expression of CD29 and CD56 and negative expression of CD31 and CD45 as previously described (Ding et al., 2018).

### Cells and Cell Culture

96-well plates (Greiner Bio-one, Netherlands) and culture flasks (Thermo Fisher Scientific, Netherlands) were coated with 0.25 μg/cm^2^ bovine collagen type I (Sigma-Aldrich, Netherlands) and incubated for at least 1 h in a humidified incubator (37°C, 5% CO_2_). Prior to use, the plates and culture flasks were washed twice with PBS. After thawing, FACS sorted satellite cells were seeded at a minimum density of 1,800 cells/cm^2^ or higher densities as indicated. For serial passaging, cells were passaged to maintain a density of <80% confluence and counted at each passage. Where indicated, bovine satellite cell differentiation was induced when at 90%–95% confluence with DMEM (1 g/L glucose) + 2% FBS.

### Cell Proliferation Assay

Myoblasts proliferated for 4–7 days. Every second day, 100% of the medium was refreshed. The cellular response was monitored by cell counting, performed with a high content analyser (ImageXpress Pico Automated Cell Imaging System) or with a proliferation assay MTS kit [Promega, CellTiter 96^®^ Aqueous One Solution Cell Proliferation Assay (MTS)]. Prior to the MTS assay, the medium was aspirated and replaced with fresh medium to avoid inaccuracies due to evaporation. Subsequently, 20 µl CellTiter 96^®^ AQueous One Solution Reagent (Promega, United States) was added to 100 µl of culture medium. The reagent was incubated for 2 h in a humidified incubator (37°C, 5% CO_2_) and measured directly with a 96-well plate reader (Viktor, PerkinElmer Life Sciences, United States) at a wavelength of 490 nm. Background correction was performed by measuring coated, medium filled wells without cells. The resulting absorption was subtracted from the absorption obtained in the experimental conditions. After execution of the MTS assay, cells were fixed with 4% paraformaldehyde. After incubation for 10 min, the cells were washed twice with PBS, followed by nuclei staining with Hoechst (1:1000 in PBS). HCA measurement was performed at x4 or x10 magnification. Unless otherwise stated, error bars indicate standard deviation. Optical microscopy (EVOS M5000, Thermo Fisher Scientific, Netherlands) was used to observe cellular morphology.

### Multipassage Assay

For the multipassage analysis, bovine satellite cells (bSCs) isolated from two different donor animals (indicated with ISO44 and ISO45) were cultured in the most effective SFM formulation and in GM (DMEM/F12 supplemented with 20% FBS and 5 ng/ml FGF-2). The cells were seeded at 3,000–4,000 cells/cm^2^ in T25 flasks and passaged every 3–5 days to maintain a confluence <80% for five passages. Every 2–3 days, the medium was fully exchanged. The flasks were also pre-coated with collagen and maintained in an incubator at 5% CO_2_ and 37°C. At each passage, bSCs were detached using either Gibco^™^ TrypLE^™^ Select Enzyme (Thermo Fisher Scientific, Netherlands), for cells grown in serum-free medium, or Gibco^™^ 0.25% Trypsin-EDTA (Thermo Fisher Scientific, Netherlands) (GM control) and counted using 0.4% trypan blue stain (Thermo Fisher Scientific, Netherlands). The number of population doublings was determined at each passage and summed up to calculate the cumulative population doublings.

To assess the maintenance of the differentiation capacity during long-term passaging in the developed animal-free formulation, cells were seeded at 40,000 cells/cm^2^ in a 12-well plate and incubated in proliferation medium until 90% confluence was reached. At that point, differentiation was induced with DMEM (1 g/L glucose) supplemented with 2% FBS. The differentiation cultures were maintained for 7 days. Optical microscopy was used to qualitatively assess differentiation.

### Statistical Analysis

For the short-term proliferation experiments, conditions were tested for significant difference using one-way ANOVA with *post-hoc* Tukey’s test in SPSS statistics (Version 27 for Mac, IBM corp., United States) unless otherwise specified. Data comparison was considered statistically significant when *p* < 0.05 (*). *p* < 0.01 was indicated with (**), *p* < 0.001 with (***) and *p* < 0.0001 (****). Non-significant results were noted as ‘‘ns’’. If not otherwise stated, error bars indicate standard deviation. For step 3, the DoE JMP^®^ statistical analysis software (SAS Institute, Inc., Cary, NC) was used to perform a two-level full factorial design combined with a stepwise regression analysis for the identification of significant factors and their combinations. Likewise, means were first compared by one-way ANOVA, followed by *post-hoc* Tukey’s test for comparison between groups using SPSS. For dose-response experiments, biological replicates refer to experiments performed on different days while technical replicates refer to concurrently run experiments within the same biological replica (if not otherwise stated, *n* = 3). GraphPad Prism 9 was used for statistical analysis and to generate figures using the HCA generated values, which were expressed as confluence (cells/mm^2^). A two-way ANOVA followed by a *post hoc* Dunnett test was performed for statistical analysis. Thereby, the mean confluence resulting for each component concentration for each time point was compared to the mean of the current component concentration, highlighted with a dashed line in each.

## Results

In previous studies, attempts to proliferate bovine myoblasts in SFM formulations that are commercially available have been reported (Kolkmann et al., 2020). Unlike these media whose exact composition is usually proprietary, the main purpose here was to develop a chemically defined SFM that supports the attachment and expansion of bovine satellite cells.

### Step 1—Starting With a Mix Containing a Basal Medium and Major Fetal Bovine Serum Components

In a first step, a comprehensive literature analysis was pursued to identify factors crucial for attachment and proliferation. The initial serum-free formulation tested was comprised of Ham’s F 10 Nutrient Mix as basal medium supplemented with the components listed on Table 2.

**TABLE 2:** Overview of the first formulation components with indication of their respective functions, final concentrations and references.

Component	Function	Concentration	References
Bovine serum albumin	major component of FBS; can bind a wide range of essential compounds such as steroid hormones, metals, and vitamins; may act as detoxifying protein	5 mg/ml	Mather (2012), Shiozuka and Kimura (2000)
Insulin	Glucose and amino acid uptake, lipogenesis, and intracellular transport; synthesis of proteins and nucleic acids	5 μg/ml	Shiozuka and Kimura (2000), Ham et al. (1988)
Transferrin	Essential trace element that binds iron and facilitates its transport into the cells; acts as detoxifying compound by removing trace amounts of toxic metals	30 μg/ml	Shiozuka and Kimura (2000), Vaheri et al. (1973), Ham et al. (1988)
Sodium Selenite	Essential trace element that acts as co-factor for glutathione peroxidase and other enzymes, thus serving as an antioxidant	2 ng/ml	Allen et al. (1985), Sheehan and Allen (1999)
Dexamethasone	Was shown to improve myogenesis *in vitro* by enhancing differentiation and myotube fusion when initially added during proliferation	0.196 μg/ml	Allen et al. (1985), Ham et al. (1988), Syverud et al. (2016)
Fetuin	Was found to be a major cell attachment factor in serum and was also shown to promote spreading and growth	500 μg/ml	Allen et al. (1985), Ham et al. (1988), Sakwe et al. (2010)
Vitronectin	Vitronectin is characterized to be a major attachment factor, that is, present in FBS; It was shown that the cell attachment activity of vitronectin was 8–16 fold greater than that of fibronectin	10 μg/ml	Hayman et al. (1985), Richards et al. (2008)
Fibronectin	Known to enhance cell attachment	10 μg/ml	Hayman et al. (1986), Richards et al. (2008)
Somatotropin (aka cow growth hormone—CGH)	It was shown that CGH, in combination with insulin, stimulated the formation of myotubes in chick embryonic myoblasts	2 ng/ml	Haba et al. (1966), Halevy et al. (1996)
α-linolenic acid (ALA)	The supplementation of growth medium with fatty acids such as ALA or oleic acid (OA) was shown to enhance satellite cell proliferation in a dose-dependent manner	1 μg/ml	Allen et al. (1985)
FGF-2	The addition of FGF-2 was shown to stimulate the proliferation of skeletal muscle cell lines while inhibiting the differentiation of these cells	5 ng/ml	Sassoon (2002), Shiozuka and Kimura (2000), Syverud et al. (2016)

### Step 2—Substitution With Animal-Free or Less Costly Homologues and Removal of Redundant Components

**FIGURE 2:**
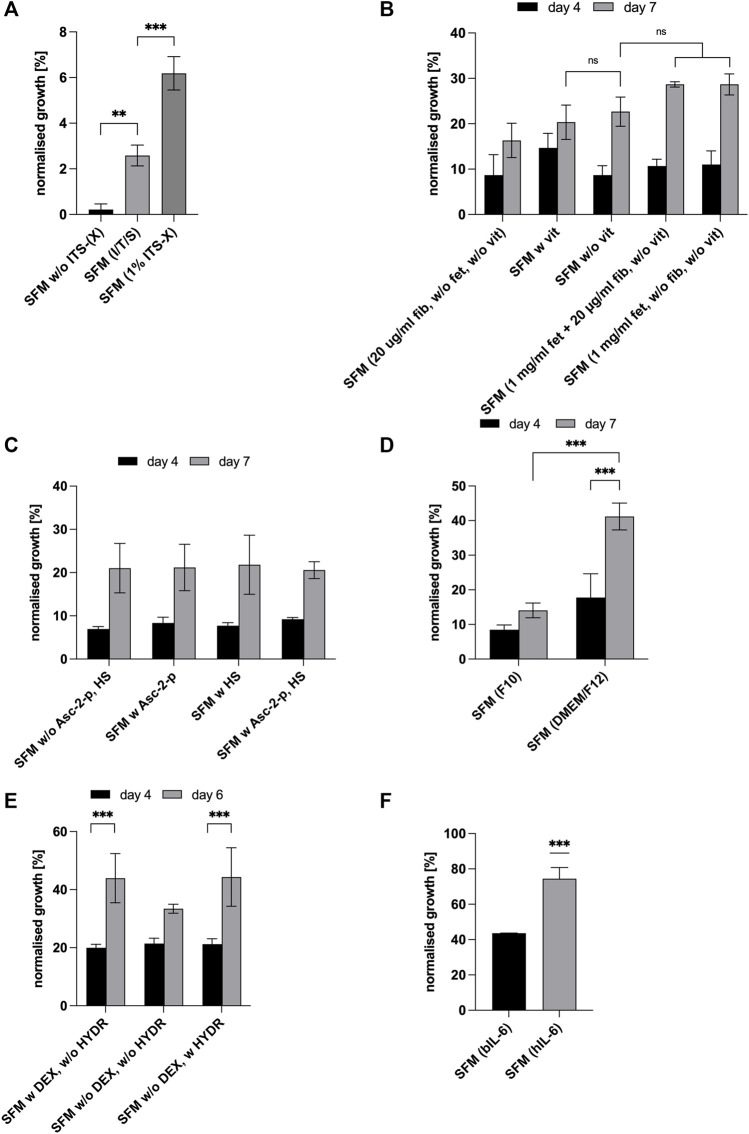
For all graphs, cell growth was measured with MTS or HCA and expressed as percentages of GM (control, not shown in graph). **(A)** Effect of the addition of insulin (I), transferrin (T) and sodium selenite (S) in comparison to supplementation of SFM with commercial Gibco^®^ ITS-X (X = Ethanolamine) at day 7 of culture. Asterisks (**p < 0.01, ***p < 0.001) indicate significant effect of the presence of ITS (-X) in the serum-free formulation [SFM w/o ITS (-X)]. **(B)** Effect of attachment factors [vitronectin (v), fibronectin (fib) and fetuin (fet)] at different concentrations on cell growth in SFM and expressed as % of growth achieved in GM (bars not shown) on days 4 and 7. **(C)** Effect of L-Ascorbate-2-phosphate and heparan sulphate on cell proliferation on days 4 and 7. For all conditions, time significantly impacted cell proliferation (*p* < 0.001). **(D)** Effect of different basal media and time, DMEM/F-12 and Ham’s F-10 Nutrient Mix on cell expansion on days 4 and 7. Asterisks (***p < 0.001) indicate that the mean value is significantly different between DMEM/F12 & F10 and that there is a significant time effect for DMEM/F12. **(E)** Effect of dexamethasone and hydrocortisone on cell proliferation on days 4 and 6. Asterisks (***p < 0.001) indicate that there is a significant time effect for—DEX + HYDR. **(F)** Comparison of bIL-6 with hIL-6 on days 4 and 6. SFM with hydrocortisone and DMEM/F12 in both cases.

The purpose of the first experiment was to investigate if the presence of insulin, transferrin and sodium selenite is necessary and if so, to compare a commercially available solution containing insulin (I), transferrin (T), sodium selenite (S) and ethanolamine (X) (Gibco^™^ ITS-X, Thermo Fisher Scientific, Netherlands) to the respective component concentrations (excluding ethanolamine) as described above and initially adopted based on the literature (Liu et al., 2019; Hu Ma et al., 2011; Mainzer et al., 2014; Chase et al., 2010). ITS-X 1% (final concentration in the medium: insulin: 10 μg/ml, transferrin: 5.5 μg/ml, sodium selenite: 6.7 ng/ml, ethanolamine: 2 μg/ml) was added to the medium as per the manufacturer’s instructions. Cells were cultured for 7 days and the number of viable cells was measured with MTS (Figure 2A) and expressed as a percentage of growth medium (positive control, graph not shown). The presence or absence of I/T/S significantly affected cell proliferation (*p* < 0.01) (Figure 2A). There have been some studies indicating that insulin might be dispensable for the growth of primary cells, however this was not confirmed here. On the contrary, this result is more consistent with results from studies using hepatocytes (Richman et al., 1976) or chick fibroblasts (Smith and Temin, 1974) where the inclusion of insulin had a significant effect on cell growth and cell cycle progression of cells that had been arrested in G_1_ (Straus, 1981). A comparison of medium containing (I/T/S) with medium containing 1% ITS-X commercial mix, shows that the addition of 1% ITS-X significantly increased cell proliferation (*p* < 0.001), indicating that either the concentrations of insulin, transferrin, and sodium selenite in the commercial mix are more effective for growing bovine myoblasts or that the addition of ethanolamine, a precursor of phosphor-glycerides and thus essential to the structure of the plasma membrane and cellular organelles, has a positive impact on cell growth (Figure 2A). Ethanolamine was shown to be an essential growth factor for hybridomas in serum-free culture (Kovář and Franěk, 1984). Based on these results, the SF medium was supplemented with 1% ITS-X for further experiments.

Subsequently, it was investigated if all the initial attachment factors included in the formulation are essential for cell attachment and growth and at which concentrations. Taking into consideration the high costs of attachment factors, especially vitronectin, and the overall aim of this study, namely the development of a simple cost-efficient and serum-free medium, the objectives of the following experiment were to determine the required factors for bSCs to attach and proliferate and whether vitronectin can be excluded. Results are shown in Figure 2B where the highest cell yields in the absence of vitronectin were achieved in the presence of fetuin (1,000 μg/ml) or in the presence of fetuin (1,000 μg/ml) and fibronectin (20 μg/ml). However, these differences were not significant when lower concentrations of fetuin and fibronectin were used (ns). Since the absence of vitronectin did not significantly reduce cell proliferation when fetuin and fibronectin were present, it was removed from the formulation. For all conditions tested, attachment of cells was unaffected (data not shown).

To optimise the serum-free medium for bovine myoblasts, the SFM was supplemented with L-Ascorbate 2-phosphate (Asc-2-p) and/or heparan sulphate (HS). Asc-2-p is a widely used medium supplement for the cultivation of stem cells and was shown to stimulate human mesenchymal stem cell proliferation without loss of phenotype and differentiation capacity (Devireddy et al., 2019). Heparan sulphate, a co-factor of FGF-2, enhanced growth of human embryonic stem cells in serum-free conditions (Furue et al., 2008) and is known to play an important role in forming the HGF-c-met complex, that is, expressed in satellite cells and myoblasts and that binds hepatocyte growth factor (HGF) which induces the activation of quiescent satellite cells *in vitro* (White and Smythe, 2016).

The absorption, measured as percentage of growth medium, was somewhat higher when Asc-2-p was added to the medium, whereas the presence of heparan sulphate seems to slightly decrease proliferation of bovine myoblasts (Figure 2C). However, no significant effect resulted from the presence of both of them (ns) while a significant time effect was observed for all conditions (*p* < 0.001). However, since both heparan sulphate and Asc-2-p may act synergistically with other (growth) factors, it was decided to include them in the formulation for the subsequent GF studies (step 3).

Subsequently, different basal media DMEM/F-12 (Barnes and Sato, 1980a) and Ham’s F-10 Nutrient Mix (Ham, 1963) commonly used in cell culture were tested for their performance. Comparing DMEM/F-12 (Barnes and Sato, 1980a) and Ham’s F-10 Nutrient Mix (Ham, 1963) (Figure 2D) showed that DMEM/F12 has a significant positive effect on the proliferation of bovine myoblasts. Based on these results, F10 was replaced by DMEM/F12 for all subsequent steps.

Dexamethasone, being a synthetic drug, would most likely raise regulatory questions if used in a formulation to support culture meat production for human consumption. For that reason, proliferation of bovine myoblasts in serum-free conditions was tested with dexamethasone being replaced by hydrocortisone, another glucocorticoid hormone (Figure 2E). It naturally occurs in FBS and is commonly used as a supplement for cell culture media as it is known to support adhesion and growth of various mammalian cells (Devireddy et al., 2019). It is a rapid and short-acting glucocorticoid whereas dexamethasone is a synthetic, long-acting one, that is, 25 times more potent (Hoofnagle et al., 2013).

Replacing dexamethasone with hydrocortisone did not significantly impact cell proliferation (Figure 2E). In both cases, 44% of growth compared to the control (GM) was reached on day 6. Thus, the SFM formulation was modified by replacing dexamethasone with hydrocortisone.

Next, we investigated whether human recombinant IL-6 is equally or more effective than the animal-derived bovine IL-6 for proliferation of bovine satellite cells. As can be observed from Figure 2F, substituting the bovine IL-6 with human recombinant one, led to a significant increase of bSCs proliferation (*p* < 0.001) resulting in 75% of growth compared to the GM. This observation is consistent with the results obtained by Brandt et al. (2018). Comparison of the bioactive peptides of IL-6 (*Bos taurus*) and IL-6 (*Homo sapiens*) through global pairwise alignment using the Needleman-Wunsch Algorithm in BLAST revealed the orthologs were 53.3% identical and 69.8% similar. The growth-enhancing effect of hIL6 could be explained by the higher affinity of species-specific inhibitor proteins suppressing the bioactivity of bIL6 in bovine satellite cells (Brandt et al., 2018). Future work should include a more detailed study of the structure and function of IL-6 molecules derived from various species. However, these investigations were not within the scope of this study.

Based on the experimental results that were outlined in this section, it was possible to further optimize the composition of our SFM towards a more economical and effective formulation.

### Step 3—Addition of Growth Factors

As outlined in the materials and methods section, several growth factors and cytokines, associated with enhanced proliferation of stem cells, were tested individually or in combination by performing a two-level full factorial design of experiments. This procedure was followed by a stepwise regression analysis in JMP^®^ to estimate all main effects of the five tested growth factors and the interactions between the factors independently in this experimental design. The aim was to identify a factor combination that matches or even outperforms the growth promoting effects of FBS. Proliferation of bovine myoblasts was determined by counting the cells on day 5, *via* HCA. Based on relevant literature references, the following growth factors and cytokines with their respective concentrations were chosen to examine their effects on growth and expansion of bovine myoblasts: IGF-1, HGF, LIF, EGF, PDGF-BB, and VEGF. The resulting experimental matrix contained 64 conditions in Supplementary Table S2 in total with an assigned value for each of the 6 growth factors (“+” = addition of GF at stated concentration; “−” = absence of respective GF).

The addition of FGF-2 to serum-free medium is known to promote the proliferation of primary cells and is already included in the SFM (Chen et al., 2014). Therefore, 5 ng/ml FGF-2 was added to all 64 conditions listed in Supplementary Table S1. High concentrations of IGF-1 from 30 to 100 ng/ml have been shown to promote proliferation while low concentrations of 1–3 ng/ml seem to promote fusion of satellite cells and thus differentiation (Rosenthal and Cheng, 1995; Engert et al., 1996; Coolican et al., 1997). More specifically, it has been reported that IGF-1 at higher concentrations induces satellite cell proliferation and promotes muscle hypertrophy (White and Smythe, 2016). For these reasons, 100 ng/ml was added to the formulation (Richards et al., 2008; Spangenburg and Booth, 2002; Czifra et al., 2006).

It is known that the heparin-binding protein HGF activates satellite cells and regulates cell proliferation activity in a dose-dependent manner (White and Smythe, 2016; Yamada et al., 2010). It has also been reported that HGF activates satellite cells by interacting with the c-met proto-oncogene receptor in quiescent satellite cells (Allen et al., 1995; Anastasi et al., 1997). HGF is shown to act synergistically with FGF-2 or PDGF (Syverud et al., 2016). The medium was thus supplemented with 5 ng/ml HGF.

The cytokine LIF is closely related to IL-6, both signal through the shared cytokine receptor gp130 (Nicola and Babon, 2015). Typically, LIF is added to inhibit spontaneous differentiation of satellite cells and to stimulate cell proliferation (White and Smythe, 2016). Here, LIF was added to a final concentration of 5 ng/ml (Spangenburg and Booth, 2002; Doumit et al., 1993).

PDGF-BB is an important mitogen that was shown to promote human MSC proliferation when being supplemented in combination with FGF-2 and TGF-β1 (Chase et al., 2010; Ng et al., 2008). To determine the importance of PDGF-BB for bovine myoblast proliferation, the serum-free formulation was enriched with 10 ng/ml PDGF-BB (Doumit et al., 1993).

In some studies, the treatment of bovine cultures with EGF resulted in the inhibition of differentiation (Allen and Boxhorn, 1987; Greene and Allen, 1991). It has been observed that EGF stimulates DNA synthesis synergistically with IGF-1 and PDGF in BALB/c-T3t3 cells (Leof et al., 1980; Pledger et al., 1982). For these reasons, the medium here was supplemented with 10 ng/ml EGF (Doumit et al., 1993; Eberli et al., 2009).

Lastly, the addition of 10 ng/ml VEGF (Germani et al., 2003; Wang et al., 2017) was tested here since it is known for its anti-apoptotic effect (Germani et al., 2003) and it was shown to increase muscle-derived stem cell proliferation in a dose-dependent manner (Wang et al., 2017). The raw data from this study can be found in Supplementary Figure S1.

**FIGURE 3:**
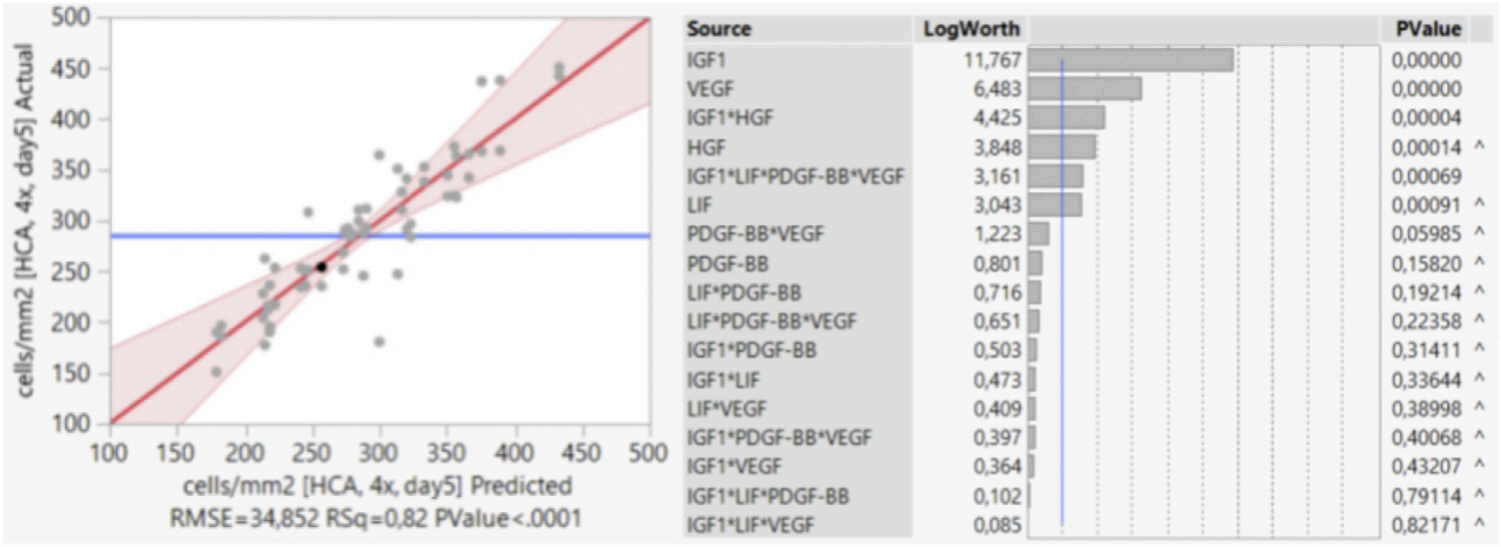
Stepwise regression analysis in JMP^®^. Scatter plot showing the actual values and the values predicted by the model. Effect summary of all factors and factor combinations that were selected by the model, ranked based on their respective *p*-values.

First, the distribution of the results of the 64 runs was monitored by histogram to confirm normal distribution of the data (data not shown). For the stepwise regression, main effects and interaction effects with *p*-values < 0.05 were considered to be significant and were incorporated into the model. As the growth factor EGF was found to not be significant and also not involved in significant interactions, it was removed during the process. The actual values and the values predicted by the model are shown in a scatter plot (Figure 3). In an effect summary (Figure 3) all significant factors and factor combinations that were selected for the model are listed and ranked based on their respective *p*-values. The analysis showed that proliferation of bSCs (in short-term) was most significantly influenced by the main effects of IGF1, VEGF, HGF, and LIF; and by the interaction effects between IGF1 and HGF and between IGF1 and LIF, PDGF-BB, VEGF. The symbol “^” indicates that a factor or a combination of factors is involved in an interaction, that is, significant although the presence of the factor or combination alone might not be significant.

**FIGURE 4:**
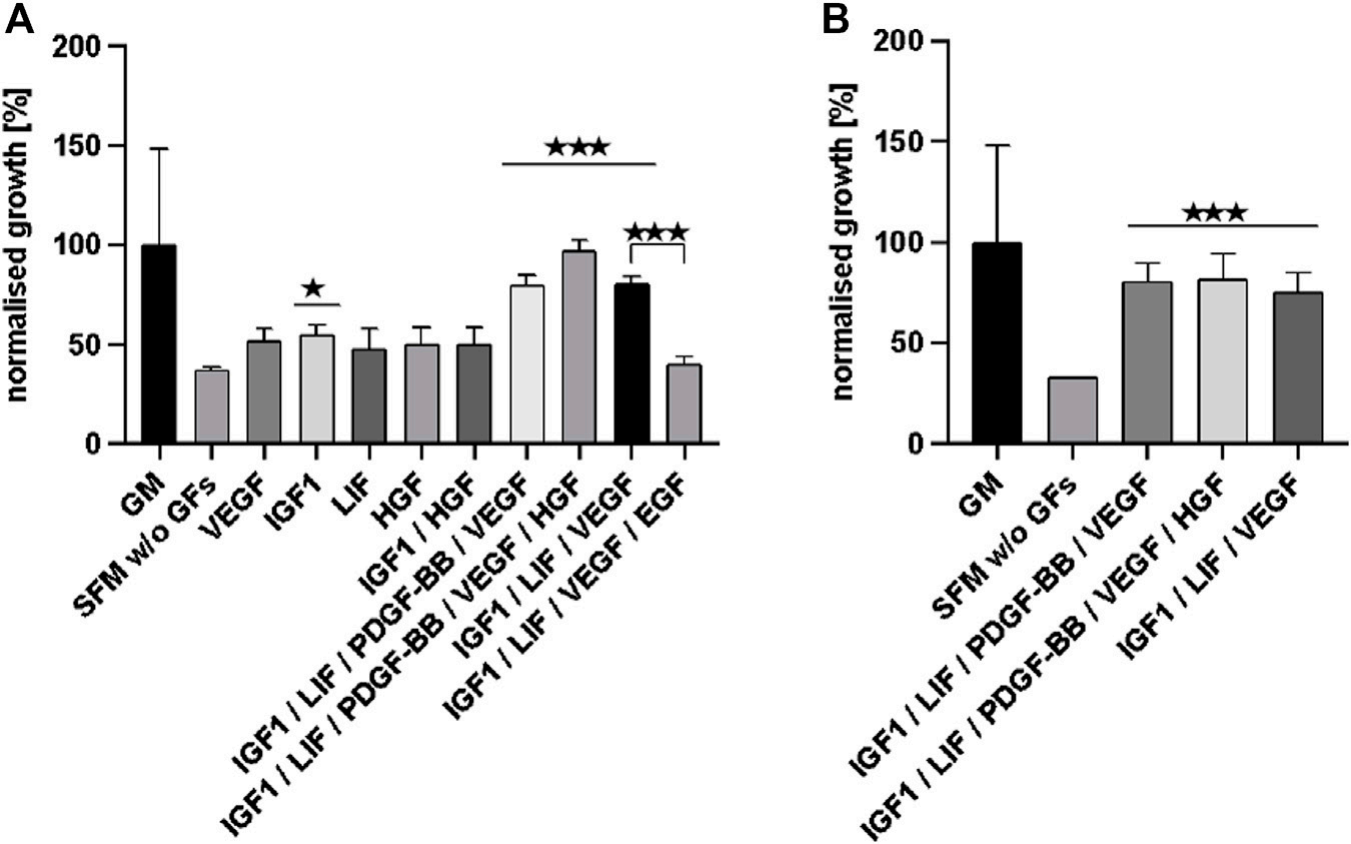
**(A)** displays the effects that significant growth factors and growth factor combinations had on cell growth (according to JMP analysis) in comparison to SFM w/o additional GFs (apart from FGF-2). Data is normalised as % of growth compared to the control (GM). The three most effective growth factor combinations were tested again and the results are shown in **(B)**.

Figure 4A displays the effects that the presence of significant growth factors and growth factor combinations had on cell growth in comparison to the SFM—not supplemented with growth factors (with the exception of FGF-2)—as normalised growth over the control (GM). The three growth factor combinations with the most significant impact (***) were then tested again for confirmation and these results are shown in Figure 4B. Comparing Figures 4A,B shows that the three different growth factor combinations reached similar values and were nearly as effective as the serum-based GM control in supporting bSCs’ proliferation.

From the results described above we can conclude that the SFM supplemented with IGF1, LIF, PDGF-BB, VEGF, and HGF supported exponential cell growth as effectively as the current FBS containing growth medium (up to 97% of GM).

However, further optimization of the concentration of individual components was deemed necessary in order to achieve a more cost-efficient formulation and/or better cell growth, leading to the experiments performed for step 4.

### Step 4—Optimisation of Concentrations

After identification of effective components, we performed dose-response analyses to find optimized component concentrations for better growth or to similar growth at reduced costs. Therefore, BSA, ITS-X, ALA, hydrocortisone, CGH, heparan sulphate, L-ascorbic acid, fibronectin, fetuin and human IL-6 were added at a higher, a lower concentration and omitted completely. As LIF and IL-6 are closely related cytokines that both signal through the shared cytokine receptor gp130 (Nicola and Babon, 2015), another purpose of this experiment was to examine if LIF and IL-6 could be mutually exchanged or whether they act in a synergistic manner.

**FIGURE 5:**
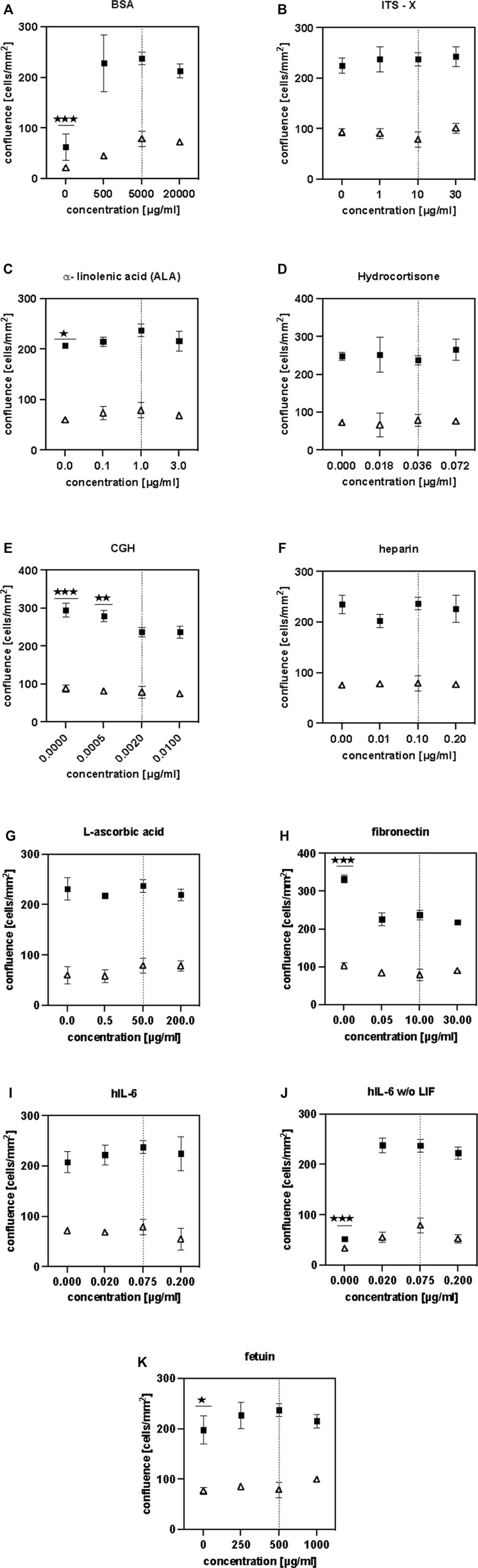
**(A–K)** illustrate the findings of the dose-response studies. HCA measurements were performed on days 3 (△) and 5 (■). Asterisks (*p < 0.05, **p < 0.01, ***p < 0.001, ****p < 0.0001) indicate significant difference compared to the control (initial component concentration, indicated with a dashed line) .

The absence of BSA resulted in a significantly reduced cell confluence on day 5 (Figure 5A). The highest confluence was reached using the control concentration of 5 mg/ml. Adjusting the ITS-X concentration did not have a significant influence on cell proliferation in short-term (Figure 5B). The absence of ALA significantly reduced cell proliferation (Figure 5C). Thus, ALA is an important component of the SFM. The addition of hydrocortisone did not have any effect on cell growth in short-term (Figure 5D). The presence of somatotropin (CGH) was found to have a significantly deleterious effect on bovine satellite cells (Figure 5E) and was therefore excluded from the SFM. Interestingly, a further increase in the concentration of CGH did not result in further significant reduction of cell growth.

Heparan sulphate does not seem to have any positive effect on proliferation (Figure 5F) as its absence did not lead to a significant reduction in cell growth. As a result, heparan sulphate was removed from the SFM formulation. The addition of L-ascorbic acid to the culture medium (Figure 5G) did not show a significant effect in short-term culture but since some sources report potential benefits in long-term culture (Furue et al., 2008), it was not removed from the SFM.

While the absence of fibronectin resulted in a significant increase of cell growth (****), the absence of fetuin lead to a significant reduction in growth. Considering the costs and availability, recombinant fibronectin is more economical and commercially available in larger quantities than recombinant fetuin. Since satellite cells are known to require attachment factors for their *in-vitro* culture it was decided to keep the fibronectin in the formulation at 10 μg/ml but to remove fetuin as it was deemed to be unsustainable for scaling up.

Modifying the concentration of hIL-6 did not have a significant effect on cell growth when LIF was present (Figure 5I). However, in the absence of LIF, it is shown that hIL-6 is necessary to sustain cell proliferation (Figure 5J). Comparing cell confluences reached in Figures 5I,J, it is evident that the presence of both cytokines did not have a cumulative effect and that the addition of hIL-6 at 20 ng/ml did not have a significant effect on cell growth when LIF was absent. We thus decided to lower the concentration of IL-6–20 ng/ml and to remove LIF from the formulation.

Summarising the findings described above, the SFM formulation was modified as follows: CGH, fetuin, LIF and heparan suphate were removed, while the concentration of hIL-6 was reduced from 75 ng/ml to 20 ng/ml (Table 1).

### Step 5—Validation in Long-Term Culture

**FIGURE 6:**
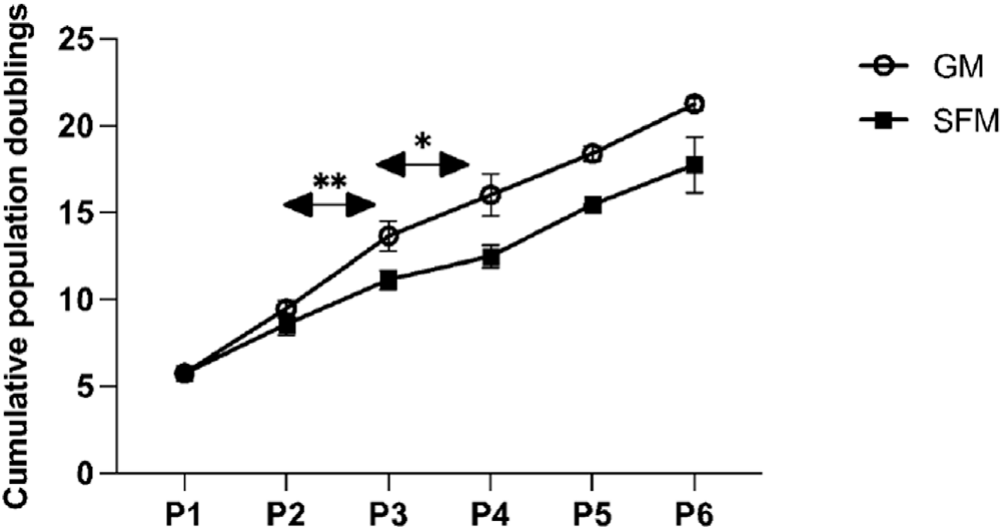
Multi-passage assay of two different donors indicated (each in duplicates) in SFM and GM. The line graph shows the means of the cumulative population doublings over the number of passages for GM and SFM. The error bars indicate standard deviations. Asterisks indicate significant differences of population doublings per passage between the two conditions. At P1 the number of population doublings is >0 due to the short pre-expansion of cells after their isolation and prior to their freezing in liquid nitrogen.

**FIGURE 7:**
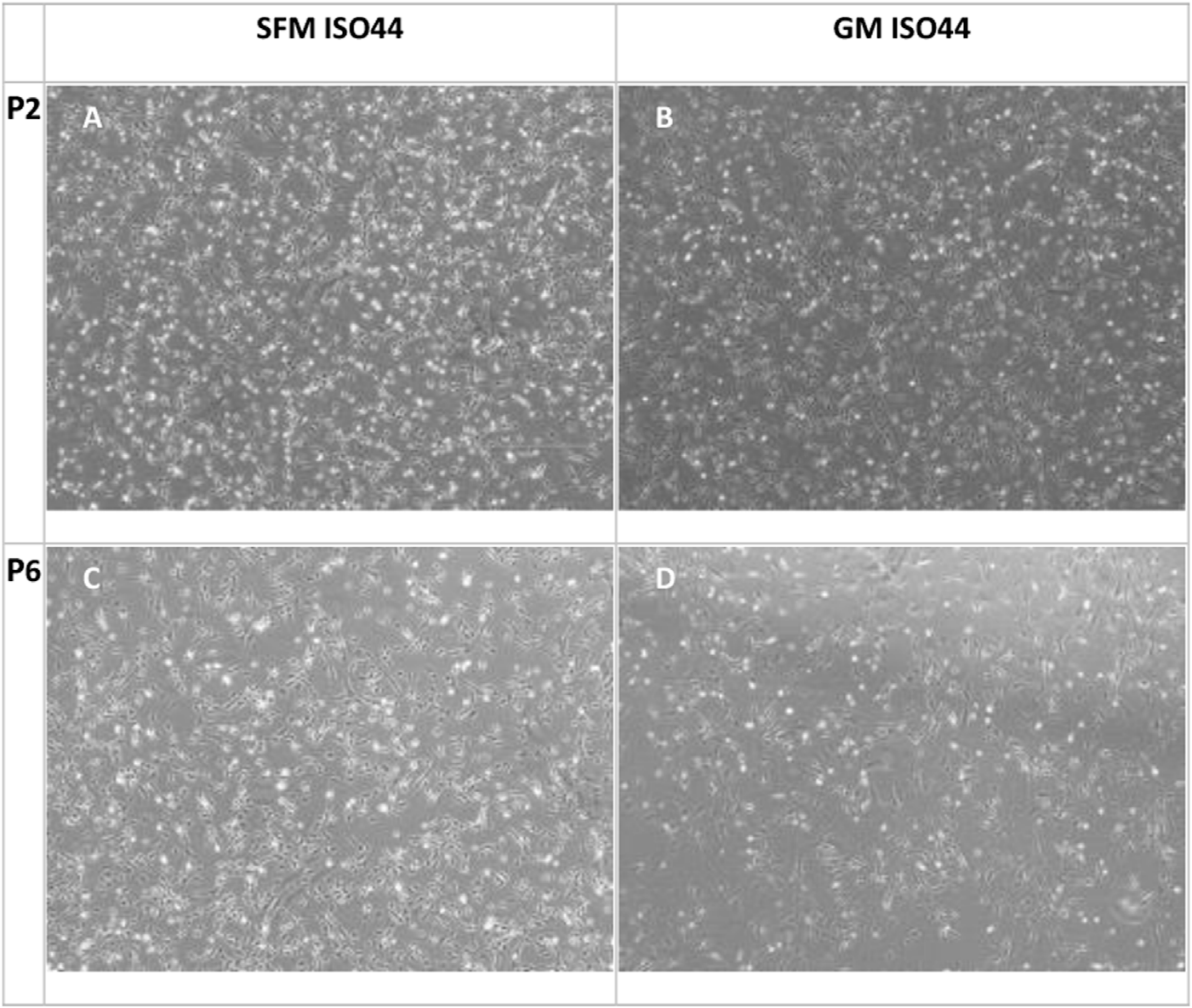
Phase contrast microscopy images of cell proliferation cultures at confluence for P2 **(A,B)** and P6 **(C,D)** with SFM and GM. In general, the cells growing in SFM show a more rounded morphology compared to GM.

**FIGURE 8:**
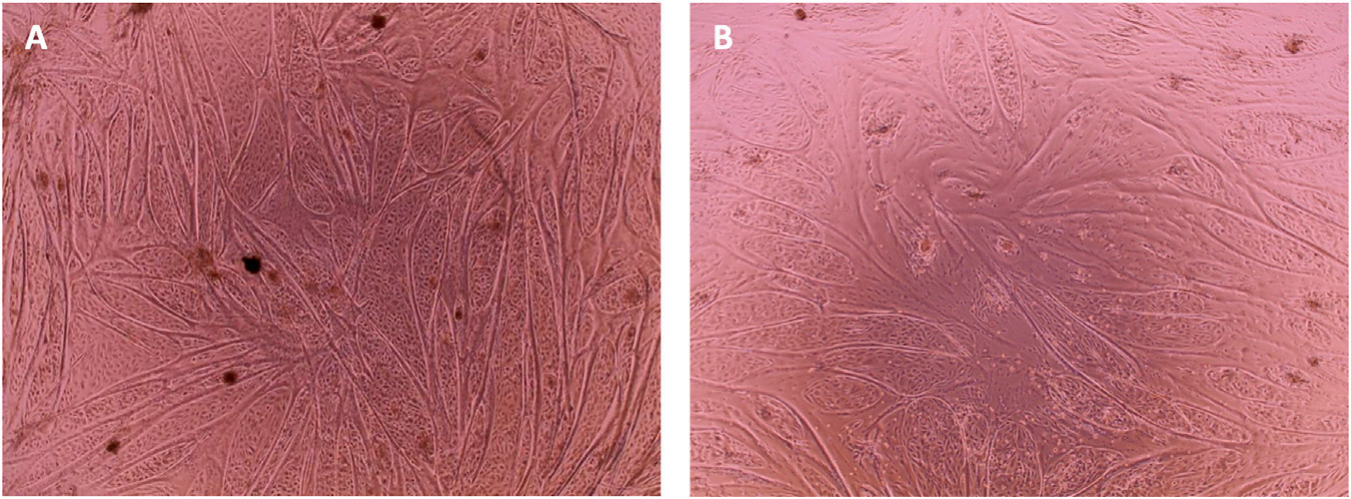
Phase contrast microscopy images of cell differentiation culture at day 7 of differentiation. Cells from ISO44 **(A)** and ISO45 **(B)** that were growing in SFM were seeded for differentiation at—16 population doublings. After 24 h in SFM, and when the cells reached a 90% confluency, differentiation was induced with DMEM (1 g/L glucose) + 2% FBS.

In order to confirm the suitability of the SFM formulation in long-term culture, a multiple passage study was performed with bSCs. For the SFM long-term culture, DMEM/F12 basal medium was supplemented with L-ascorbic acid, fibronectin, hydrocortisone, GlutaMAX^™^, HSA, ITS-X, hIL-6, ALA, FGF-2, VEGF, IGF-1, HGF, and PDGF-BB. Serum—containing growth medium was used as a control. Unlike the short-term experiments, where medium was exchanged every 2 days, for long-term passaging, medium was exchanged every 3–4 days. For this reason, the concentration of FGF-2 was doubled to a final concentration of 10 ng/ml to compensate for its degradation during the culture, as it is known to be thermally unstable at 37°C (Koledova et al., 2019). Cells from two different donors were cultured for five successive passages in duplicates and were then differentiated using the protocol as described in the materials and methods section. Using cell count data collected at each passage, cumulative population doublings (Figure 6) were calculated. In addition, brightfield images were taken to demonstrate cell morphologies and differentiation capacity (Figures 7, 8).

Similar to the findings from short-term proliferation studies, bSCs expanded robustly in our serum-free medium formulation throughout the long-term culture, with comparable numbers of population doublings per passage during P1, P3 and P4 (ns). During P2 and P3 bSCs cultured in GM surpassed the ones cultured SFM in terms of growth rate, with a significant rise in number of population doublings per passage [*p* < 0.01 (P2), *p* < 0.05 (P3)].

As can be observed from the microscopy images that were taken on the day of harvesting (Figure 7) cells growing in SFM showed a smaller and less elongated morphology compared to cells growing in GM. Given that bCSs need to differentiate into myotubes for further processing towards cultured meat, it is crucial that the serum-free proliferation formulation supports the ability of the expanded cells to differentiate effectively into bovine myotubes. Cells from both donors were successfully expanded and differentiated into bovine myoblasts (Figure 8) after being cultured for five passages in SFM. No differences in differentiation capacity were observed between the two donors. Further data on differentiation, can be found in the study by Messmer et al, where it was shown that satellite cells can be proliferated in the hereby developed proliferation medium and subsequently differentiated successfully in both serum-based and serum-free differentiation media formulations (Messmer et al., 2022).

## Discussion

Prior work has focused on the development of serum-free media formulations suitable for human cell types due to their high demand in the therapeutic field. Nevertheless, these studies mainly tested the compatibility of serum-free media for cell lines which are less serum-dependent than primary cells like bovine myoblasts. Studies on serum-free media for primary cells are limited due to challenges related to cost and the difficulty in finding a serum-free formulation, that is, precisely adjusted to the needs of the cells, in terms of composition and factor concentrations. Kolkmann et al. (2020) have successfully grown bovine myoblasts in commercially available serum-free media formulations but with the use of some animal components that are not fully defined (Kolkmann et al., 2020).

To date, no defined serum-free media formulations specific for bovine myoblasts are reported. The overall goal of this study was to develop a chemically defined serum-free medium that supports the attachment and expansion of bovine myoblasts while maintaining the differentiation capacity of the cells in order to achieve a maximum number of differentiated muscle fibers derived from a minimal tissue sample size.

In this study, the steps for developing a fully chemically defined serum free and animal free medium for primary bovine satellite cells are described. Our aim was thereby to demonstrate that satellite cells cultured in a defined animal-free medium can reach the same final number of cells compared to cells growing in a FBS-based medium. Different basal media, various components and growth factors associated with enhanced proliferation of mammalian cells were tested in short-term and in different concentrations as serum replacing supplements and growth factors in a systematic manner. The most effective formulation was then validated in long-term culture over six passages with subsequent differentiation.

Our findings are in accordance with previous results that the addition of EGF did not induce myoblast proliferation (Kolkmann et al., 2020). Moreover, the addition of EGF was found to negatively impact bSCs expansion. Surprisingly, replacing bovine recombinant IL-6 with human recombinant IL-6 not only led to an overall cost reduction of 80% but also significantly increased the proliferation of bovine myoblasts. Hammacher et al. (1994) investigated the structural similarities and differences of human and mouse IL-6 polypeptides and identified two regions that are important for receptor binding (Hammacher et al., 1994). The structure of human IL-6 may lead to a stronger receptor binding in bovine myoblasts resulting in an increased receptor activity promoting cell growth. Unlike reported by Haba et al. (1966) for chick myoblasts, somatotropin did not have any growth promoting effect on our cells (Haba et al., 1966). In contrast, it seems to be deleterious in a dose dependent manner. Surprisingly, the addition of heparan sulphate did not show a growth enhancing effect in short-term culture although it has been previously reported in the literature as being important for FGF signalling and stabilization (Harmer, 2006; Forsten-Williams et al., 2005).

In summary, the SFM formulation consisting of DMEM/F12 as basal medium and supplemented with L-ascorbic acid, fibronectin, hydrocortisone, GlutaMAX^™^, HSA, ITS-X, hIL-6, ALA and growth factors such as FGF-2, VEGF, IGF-1, HGF, and PDGF-BB most effectively supported myoblast proliferation. With this, we have developed a serum-free, chemically defined, medium for proliferation of bovine SCs that brings the development and upscaling of cultured meat production a step closer.

Continuing research could be focused on performing more advanced methods for media optimization reducing the experimental workload and thus increasing the throughput. Recently, Zhang et al. (2007) applied stochastic methods in combination with mathematical networks to predict and store information about component effects and interaction effects (Zhang et al., 2007). In addition to usual DoE techniques that provide information about which of the chosen component levels is significantly more effective, stochastic algorithms can suggest new optimal component concentrations resulting in more fine-tuned media with less experimental effort.

## Data Availability

The original contributions presented in the study are included in the article/Supplementary Material, further inquiries can be directed to the corresponding authors.
