# Supplementary material for: Development of a Chemically Defined Medium for in vitro Expansion of Primary Bovine Satellite Cells
Source: Front Bioeng Biotechnol. 2022 Aug 4;10:895289. doi: 10.3389/fbioe.2022.895289 (PMC9385969; doi:10.3389/fbioe.2022.895289)
Supplement: Supplementary file 1 [file DataSheet1.PDF]

Supplementary material

Table 1 List of serum-free media components with information on suppliers, final concentrations and source (animal-derived/recombinant).

**Component**	**Supplier**	**Final concentration**	**Animal-derived?**
Ham's F10	Thermo Fisher	-	no
DMEM/F-12	Thermo Fisher		no
ITS-X	Thermo Fisher	1%	no
GlutaMAX™	Thermo Fisher	1%	no
BSA	Sigma Aldrich	-	yes
HSA	Richcore	5 mg/ml	no
Rec. human fibronectin	Sigma Aldrich	-	no
Fibronectin	Sigma Aldrich	10 µg/ml	yes
Vitronectin	R&D systems	-	yes
Fetuin	Sigma Aldrich	-	yes
Dexamethasone	Sigma Aldrich	-	no
Hydrocortisone	Sigma Aldrich	36 ng/ml	no
Bovine IL-6	Thermo Fisher	-	no
Human IL-6	Peprotech	20 ng/ml	no
Somatotropin	Abcam	-	no
α-linolenic acid	Sigma Aldrich	1 µg/ml	no
L-Ascorbate-2-phosphate	Sigma Aldrich	50 µg/ml	no
Heparan Sulphate	STEMCELL technologies	-	yes
FGF-2	R&D systems	10 ng/ml	No
VEGF	R&D systems	10 ng/ml	No
HGF	R&D systems	5 ng/ml	No
IGF-1	R&D systems	100 ng/ml	No
LIF	R&D systems	-	No
EGF	R&D systems	-	No

PDGF-BB	R&D systems	10 ng/ml	No
Insulin	Sigma Aldrich	-	yes
Sodium selenite	Sigma Aldrich	-	No
Transferrin	Sigma Aldrich	-	No

Table 2 Experimental matrix containing 64 conditions in total with an assigned value for each of the 6 growth factors (+ = addition of GF at stated concentration; - = no addition of respective GF). Set-up for a full-factorial Design of Experiments.

Run	IGF 1	HGF	LIF	PDGF-BB	EGF	VEGF
1	+	-	-	+	+	+
2	+	+	-	+	+	+
3	-	-	-	+	+	+
4	-	+	-	+	+	+
5	+	-	+	+	+	+
6	+	+	+	+	+	+
7	-	-	+	+	+	+
8	-	+	+	+	+	+
9	+	-	-	-	+	+
10	+	+	-	-	+	+
11	-	-	-	-	+	+
12	-	+	-	-	+	+
13	+	-	+	-	+	+
14	+	+	+	-	+	+
15	-	-	+	-	+	+
16	-	+	+	-	+	+
17	+	-	-	+	-	+
18	+	+	-	+	-	+
19	-	-	-	+	-	+
20	-	+	-	+	-	+
21	+	-	+	+	-	+
22	+	+	+	+	-	+

23	-	-	+	+	-	+
24	-	+	+	+	-	+
25	+	-	-	-	-	+
26	+	+	-	-	-	+
27	-	-	-	-	-	+
28	-	+	-	-	-	+
29	+	-	+	-	-	+
30	+	+	+	-	-	+
31	-	-	+	-	-	+
32	-	+	+	-	-	+
33	+	-	-	+	+	-
34	+	+	-	+	+	-
35	-	-	-	+	+	-
36	-	+	-	+	+	-
37	+	-	+	+	+	-
38	+	+	+	+	+	-
39	-	-	+	+	+	-
40	-	+	+	+	+	-
41	+	-	-	-	+	-
42	+	+	-	-	+	-
43	-	-	-	-	+	-
44	-	+	-	-	+	-
45	+	-	+	-	+	-
46	+	+	+	-	+	-
47	-	-	+	-	+	-
48	-	+	+	-	+	-
49	+	-	-	-	-	-
50	+	+	-	-	-	-
51	-	-	-	-	-	-
52	-	+	-	-	-	-
53	+	-	+	-	-	-

54	+	+	+	-	-	-
55	-	-	+	-	-	-
56	-	+	+	-	-	-
57	+	-	-	+	-	-
58	+	+	-	+	-	-
59	-	-	-	+	-	-
60	-	+	-	+	-	-
61	+	-	+	+	-	-
62	+	+	+	+	-	-
63	-	-	+	+	-	-
64	-	+	+	+	-	-

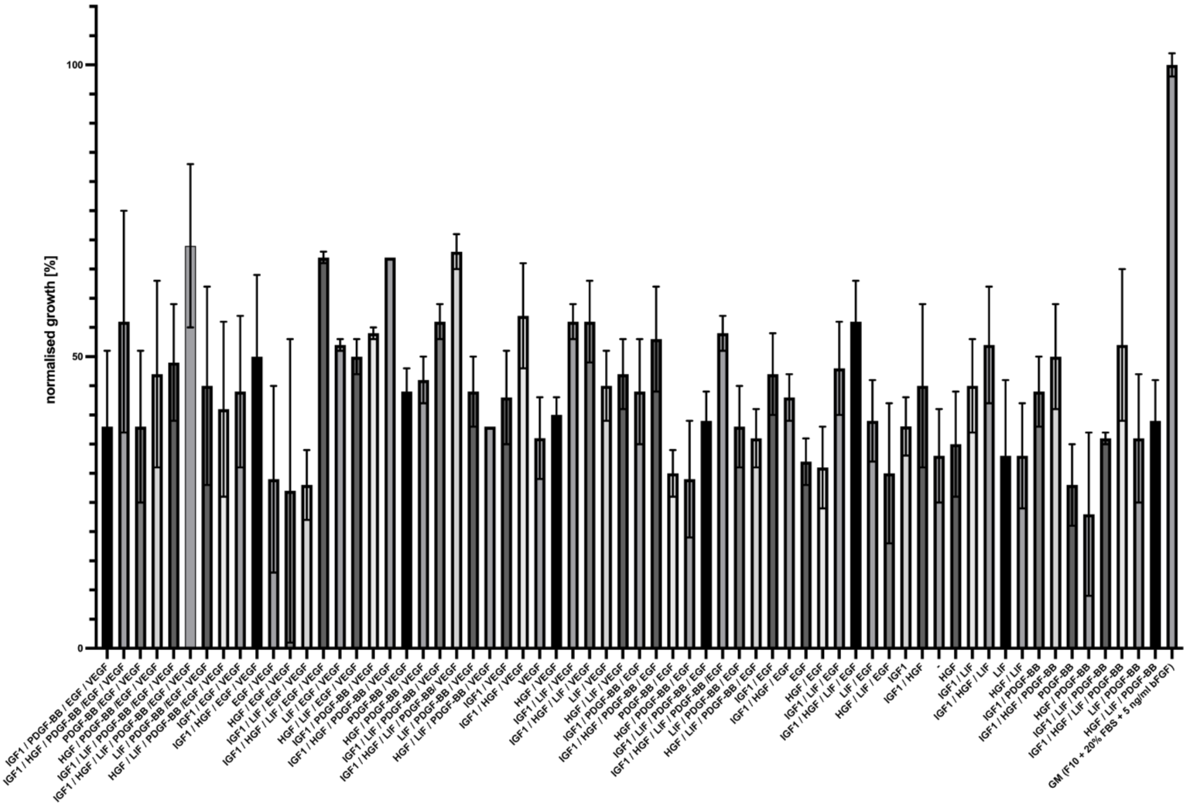

Fig.1 Day 5 cell growth data from the 2-level full factorial experimental design implemented for “Step 3 – Growth factor addition”

Table 3: Abbreviations

Fetal Bovine Serum	FBS
High Content Analyzer	HCA
L-Ascorbate-2-Phosphate / L-ascorbic acid	Asc-2-p
Heparan sulphate	HS
Human Serum Albumin	HSA
Bovine Serum Albumin	BSA
human Interleukin-6	hIL-6
bovine Interleukin-6	bIL-6
Basis Fibroblast Growth Factor	FGF-2
α-linolenic acid	ALA
Vascular Endothelial Growth Factor	VEGF
Insulin-like Growth Factor 1	IGF-1
Hepatocyte Growth Factor	HGF
Leukemia Inhibitory Factor	LIF
Platelet-derived Growth Factor BB	PDGF-BB
Transforming Growth Factor beta	TGF-β
Growth medium	GM
BaseMix	BM
Phosphate buffered saline	PBS
Fluorescence-activated cell sorting	FACS
Somatotropin / Cow Growth Hormone	CGH
Fetuin	fet
Fibronectin	fib
Vitronectin	vit
Dexamethasone	DEX
Growth Factor	GF
Hydrocortisone	HYDR
With	w
Without	w/o
